# Spinal Chloroma – Herald of blast crisis in a patient with chronic myeloid leukaemia: A case report

**DOI:** 10.4102/sajr.v26i1.2286

**Published:** 2022-01-27

**Authors:** Stuti Chandola, M. Sarthak Swarup, Radhika Batra, Alpana Manchanda

**Affiliations:** 1 Department of Radiodiagnosis, Maulana Azad Medical College, New Delhi, India

**Keywords:** myeloid sarcoma, chronic myeloid leukaemia, blast crises, acute myeloid leukaemia, chloroma

## Abstract

Myeloid sarcoma or chloroma is a localised tumour characterised by extramedullary proliferation of precursor myeloid cells. Commonly occurring in association with acute myeloid leukaemia, chloroma can occasionally be seen in myeloproliferative disorders with subsequent blastic transformation. Imaging plays an important role in the diagnosis and evaluation of this entity. A case of chloroma involving the dorso-lumbar vertebral region is presented in a patient with chronic myeloid leukaemia with subsequent blastic transformation.

## Introduction

Myeloid sarcoma, also known as granulocytic sarcoma or chloroma, refers to a solid tumour representing extramedullary proliferation of precursor myeloid cells.^1^ It affects children more commonly than adults and is equally seen in both sexes. Chloroma most commonly occurs in association with acute myeloid leukaemia (AML); however, it can also be seen in myeloproliferative disorders like polycythaemia vera and chronic myeloid leukaemia (CML) and in myelodysplastic syndromes.^2^ Although the lesion largely occurs in the active phase of AML, it can also arise when the disease is in remission. The appearance of chloroma may indicate relapse or herald a blast crisis, especially in myeloproliferative disorders.

Bones, including the spine, are typically involved because of direct extension from the affected marrow.^3^ The most common bones to be affected include the appendicular bones and spine with near equal incidence being reported in both.^1,3^ Occasionally, there can be further extension of the lesion into the spinal canal and along the nerve roots with subsequent compressive myelopathy.

A case of chloroma involving the dorso-lumbar vertebral region with associated involvement of the epidural space in a patient with CML is presented. Although the epidural region is a common location for chloroma, this disease entity itself is uncommon and occurs mostly in association with AML. The presentation of chloroma in the setting of CML with ultimate blastic conversion is a relatively rare phenomenon.

## Case presentation

A 35-year-old gentleman presented with progressive paraparesis over 15 days. He was known with chronic myeloid leukaemia (BCR-AML gene positive) and in remission for four years on imatinib therapy. Laboratory findings revealed anaemia with a haemoglobin of 8 g/dL. Other laboratory tests were normal and bone marrow aspirate did not reveal blast cells. Magnetic resonance imaging (MRI) of the spine was performed on a 3T scanner (Siemens Magnetom skyra) in view of the complaints.

**FIGURE 1:**
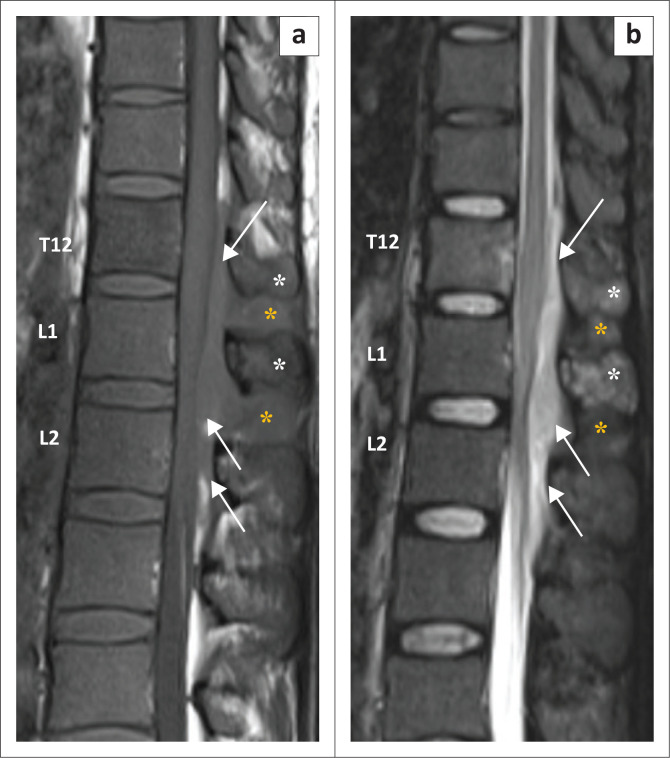
Sagittal T1W (a) and short tau inversion recovery (STIR) sequence (b) images reveal altered signal intensity involving the T12 vertebral body and spinous processes of the T12 and L1 vertebrae (white asterisks, a and b) which appear hypointense on T1W and hyperintense on STIR images. An ill-defined soft tissue mass of similar signal intensity is seen in the posterior epidural space extending from the T12 to L2 vertebral levels (arrows) with further extension into the interspinous soft tissue (yellow asterisks, a and b). T1W, T1 weighted; T12, thoracic vertebra 12; L1, lumbar vertebra 1; L2, lumbar vertebra 2.

**FIGURE 2:**
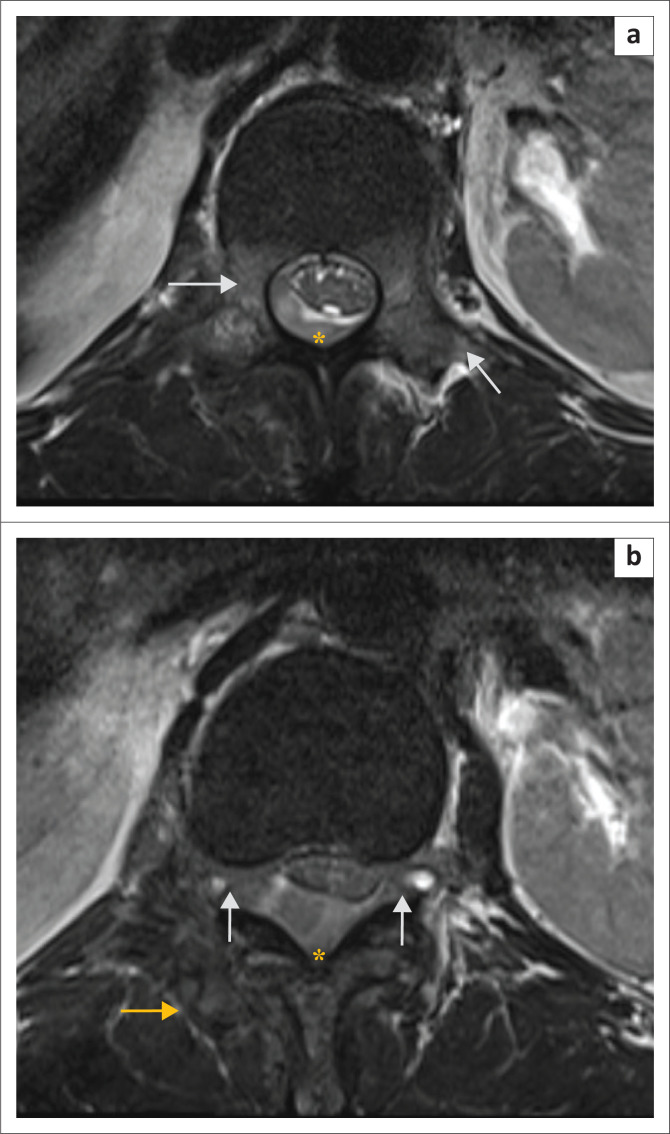
Serial axial T2W images (a and b) at the level of the L1 vertebra show hyperintense signal in both pedicles and transverse processes (arrows, a) indicating involvement. The soft tissue mass (yellow asterisk, a and b) is causing anterior displacement of the conus medullaris with marked compression (b). There is extension through and widening of bilateral neural foramina (arrows, b). The paraspinal muscles on the right side appear heterogeneously hyperintense suggestive of infiltration (yellow arrow, b). T2W, T2 weighted; L1, lumbar vertebra 1.

**FIGURE 3:**
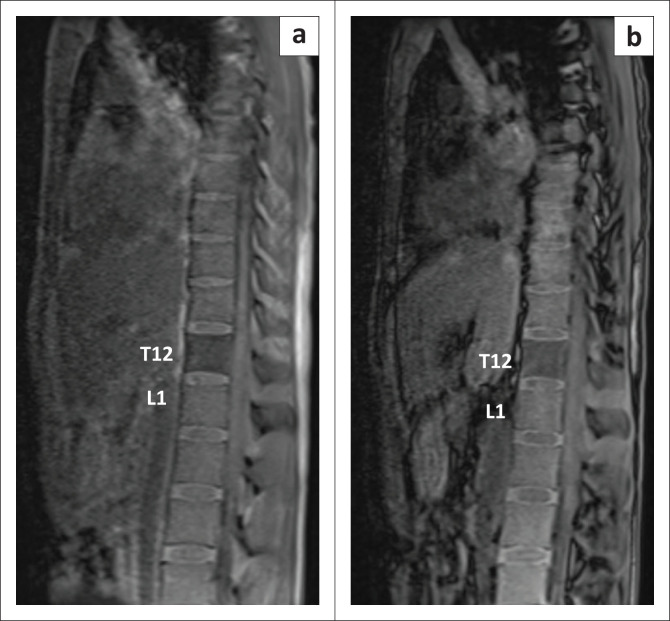
Sagittal T1W sequences acquired in phase (a) and opposed phase (b) show that the involved T12 vertebral body does not show reduction in signal on opposed phase images. This finding suggests a bone marrow infiltrating disorder as opposed to red marrow conversion, where signal reduction is expected due to presence of microscopic fat in red marrow. T1W, T1 weighted; T12, thoracic vertebra 12.

**FIGURE 4:**
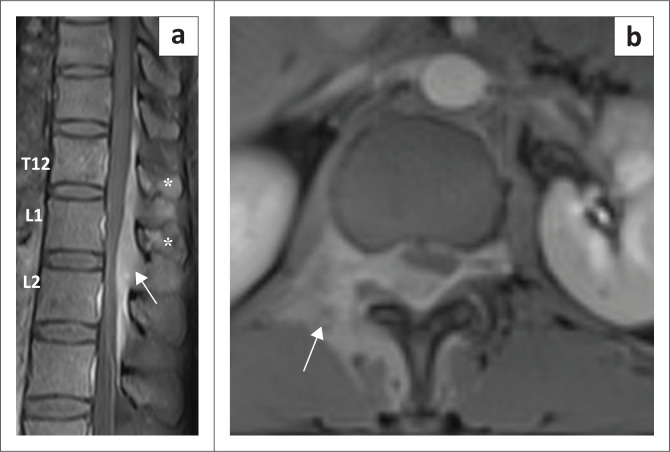
Sagittal (a) and axial (b) post contrast images demonstrate mild enhancement of the involved vertebral body (T12) and posterior elements (asterisks, a) with avid homogeneous enhancement of the infiltrative soft tissue (arrows). T12, thoracic vertebra 12.

The MRI examination revealed altered signal intensity involving the bodies and posterior elements of the T12 vertebra and L1 vertebra which showed hypointense signal on T1 weighted (T1W) and hyperintense signal on T2W and short tau inversion recovery (STIR) sequences (Figures 1 and 2) with mild enhancement on post contrast images. On chemical shift imaging, the involved vertebral body did not show reduction in signal on opposed phase images (Figure 3). There was a homogeneously enhancing soft tissue mass in the posterior epidural space extending from the T12 to L2 vertebral levels appearing isointense on T1W and hyperintense on T2W and STIR images (Figures 2 and 4). The lesion caused narrowing of the spinal canal with marked compression of the conus medullaris and cauda equina nerve roots. There was associated widening of bilateral neural foramina (right > left) at the T12–L1 and L1–L2 levels, with infiltration of the soft tissue lesion into bilateral paraspinal muscles. A possibility of myeloid sarcoma was suggested given the clinical profile and imaging findings.

**FIGURE 5:**
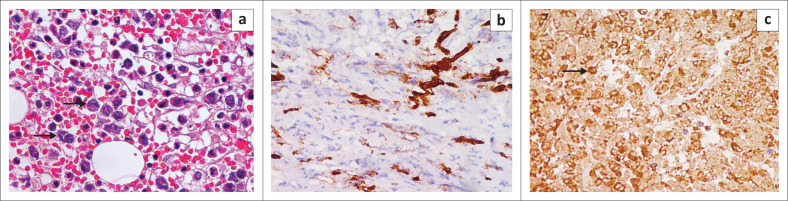
(a) The epidural lesion was subsequently biopsied and subjected to microscopic examination. High power field microscopic examination (100× magnification) with hematoxylin and eosin stain shows multiple myeloblasts (arrows) revealing large oval nuclei with a high nucleus:cytoplasmic ratio and prominent nucleoli with intermingled myelocytes showing abundant eosinophillic cytoplasm. High power field examination (100× magnification with special stains reveals that the neoplastic cells show strong positivity for CD34 (b - brown cells) and also stain positive for myeloperoxidase (arrow, c) suggestive of myeloid lineage thereby confirming the diagnosis of granulocytic sarcoma.

The patient underwent decompressive surgery with a laminectomy and debulking of the epidural lesion. Microscopic examination of the tissue showed multiple myeloblasts with large oval nuclei and prominent nucleoli scattered in a background of myelocytes (Figure 4). In addition, the neoplastic cells showed strong positivity for CD34 and myeloperoxidase enzyme (Figure 5) suggestive of myeloid lineage which confirmed the diagnosis of a granulocytic sarcoma.

The patient was then started on a combination of imatinib and steroids. However, the patient progressed into a blast crisis and full-blown AML (blast cells in bone marrow > 20%) in a span of four weeks.

Despite an initial minimal improvement with steroids, no further change was seen in the paraparesis. The patient developed severe pancytopenia with hospital acquired pneumonia and succumbed to his illness two months later.

## Discussion

First described by Burns in 1811, myeloid sarcoma or chloroma is characterised by the presence of myeloperoxidase enzyme which imparts a greenish tint to it, a feature seen in two thirds of cases. At immunohistochemical staining, it is characterised by the expression of CD68, lysozyme, CD34, and CD43 with variable expression of myeloperoxidase enzyme. Children are affected more commonly than adults and 60% of the patients are younger than 15-years of age.^1^

Clinically, chloroma most commonly occurs in the active phase of AML, particularly the M4 and M5 subtypes. However, it can occasionally occur in the setting of chronic myeloproliferative disorders (including chronic idiopathic myelofibrosis, essential thrombocythemia, polycythaemia vera and CML) or myelodysplastic syndrome, with the appearance of the tumour often corresponding with blastic conversion.^2^ In addition, chloroma may also be a manifestation of relapse in formerly treated patients of primary or secondary acute leukaemia, especially post bone marrow transplantation. In a small number of cases, it occurs in patients without any manifestations of acute leukaemia and is later heralded by the appearance of AML after an interval of weeks, months, or even years. Isolated chloromas without the evidence of leukaemia are frequently misdiagnosed because of the lack of typical CT or MR findings and need for biopsy.^4^

The cortical bone and periosteum are typically involved due to the direct spread from the affected marrow. Although any site may be involved, other common areas of involvement include the skin, lymph nodes, orbit, muscles, central nervous system, head and neck and retroperitoneum.^3^ In addition, the peritoneum and adrenals have also been documented as unusual sites.^5^

In the spine, the most commonly affected site includes the vertebral body. In addition, there can be spread of myeloid cells from the haversian canals into the extra-osseous region with resultant involvement of the epidural space, which is the most frequent area affected.^4^ It may appear similar to spinal tumours when the tumour tracks along the paraspinal nerve roots. Pre-/paravertebral chloroma may also result as an extension from retroperitoneal lymphadenopathy.

A precise and prompt diagnosis of chloroma is crucial as it allows the institution of definitive radiotherapy or adjuvant chemotherapy for local control of the disease, which helps in relieving cord compression and obviates any unnecessary surgery. Magnetic resonance imaging forms the cornerstone in the evaluation and diagnosis of spinal involvement in chloroma by aiding in spatial localisation (i.e. vertebral, epidural, intradural extramedullary and intramedullary), determining its extent and assessing the degree of cord compression, if present. At MRI, vertebral involvement in chloroma manifests as focal, multifocal or diffuse leukaemic infiltration of the vertebral marrow with low signal intensity on T1W, high signal intensity on T2W images and avid enhancement on post contrast images. Chemical shift imaging remains a useful entity when differentiating this condition from red marrow conversion. A propensity for ligamentous and subperiosteal involvement is also seen as a result of spread via the haversian canals.^6^ However, this non-specific appearance can be confused with marrow infiltrating disorders such as multiple myeloma and lymphoma; hence, a thorough clinical history of haematological disease is imperative in establishing the diagnosis.

Epidural involvement in chloroma can often occur in conjunction with vertebral body involvement. These often present as multiple masses which may be either contiguous or discrete, involving different vertebral levels. They usually show isointense and intermediate signal on T1W and T2W images respectively, with homogeneous post contrast enhancement.^7,8^ Central non-enhancing necrotic areas may be identified and diffusion restriction may be noted on diffusion weighted images. The lesions can cause effacement of the epidural fat with inward displacement of the dura mater and compression of the spinal cord with partial or complete obliteration of the cerebrospinal fluid (CSF) space. Frequent association with compressive myelopathy or neuronopathy, regardless of lesion location, is also seen. The combined intra-extraspinal form of chloroma, with a resultant ‘dumb bell configuration’, may simulate the appearance of a nerve sheath tumour on imaging. In such scenarios, absence of intense T2 hyperintensity without secondary bony changes (destruction or remodelling) favours chloroma.^4^

Intramedullary chloroma is a relatively rare entity which manifests as diffuse expansion of the cord and shows low signal intensity on T1W and high signal intensity on T2W with homogeneous post contrast enhancement. There may also be associated leptomeningeal enhancement. The appearance is non-specific and biopsy is required for final diagnosis.

Extranodal lymphoma remains an important differential diagnosis of chloroma due to similar imaging findings. It typically shows low to intermediate signal intensity on T1W images and mild hyperintense signal on T2W images with restricted diffusion on diffusion weighted sequences. Extraosseous myeloma may also mimic chloroma. This appears as a homogeneous soft-tissue mass on CT and shows low signal on T2W images. These entities may be indistinguishable from chloroma on the basis of imaging features alone and a sound knowledge of the clinical and haematological profile helps in arriving at an accurate diagnosis.

It is therefore critical for the radiologist to consider the possibility of chloroma as a differential diagnosis especially in the setting of leukaemia and other myeloproliferative disorders. The appearance of a new mass almost anywhere in the body of a leukaemic patient should raise concern for chloroma and accordingly histologic diagnosis should be sought. The diagnosis remains challenging in patients without a history of hematologic malignancy.

## Conclusion

Although commonly seen in AML, chloroma is a disease which can occur in association with a myriad of myeloproliferative and myelodisplastic disorders and is often the first manifestation of the progression of these disorders into AML (blast crisis) as seen in the presented case. Spinal involvement in chloroma may manifest as osseous infiltration with or without spinal cord involvement and is often associated with compressive myelopathy. Although the disease is associated with a poor prognosis and is treated by systemic chemotherapy, irrespective of the site or marrow status, a localised treatment plan consisting of focused radiotherapy and surgery may be formulated for relieving compressive symptoms.

Radiologists play a key role in suggesting the possibility of this disease entity, which calls for the need to familiarise oneself with the imaging features and to have a high index of suspicion, especially in patients with a history of hematologic disease. A combined multidisciplinary approach consisting of clinical features, imaging and pathological findings, including immunohistochemistry, is imperative for correct diagnosis.
